# Supplementary material for: Morphological Electrical and Hardness Characterization of Carbon Nanotube-Reinforced Thermoplastic Polyurethane (TPU) Nanocomposite Plates
Source: Molecules. 2023 Apr 20;28(8):3598. doi: 10.3390/molecules28083598 (PMC10145542; doi:10.3390/molecules28083598)
Supplement: Supplementary file 1 [file molecules-28-03598-s001.zip › molecules-2020492-supplementary.pdf]

molecules

Supplementary Materials

Morphological Electrical and Hardness Characterization of Carbon Nanotube-Reinforced Thermoplastic Polyurethane (TPU) Nanocomposite Plates

José Muñoz-Chilito [1], José A. Lara-Ramos [1], Lorena Marín [2,3], Fiderman Machuca-Martínez [2,4], Juan P. Correa-Aguirre [5], Miguel A. Hidalgo-Salazar [5], Serafín García-Navarro [6], Luis Roca-Blay [6], Luis A. Rodríguez [1,2], Edgar Mosquera-Vargas [1,2,*] and Jesús E. Diosa [1,2,*]

[1] Grupo de Transiciones de Fase y Materiales Funcionales, Departamento de Física, Universidad del Valle, Santiago de Cali 760032, Colombia
[2] Centro de Excelencia en Nuevos Materiales (CENM), Universidad del Valle, Santiago de Cali 760032, Colombia
[3] Grupo de Películas Delgadas, Universidad del Valle, Santiago de Cali 760032, Colombia
[4] Grupo de Investigación en Procesos Avanzados para Tratamientos biológicos y Químicos, Escuela de Ingeniería Química, Universidad del Valle, Santiago de Cali 760032, Colombia
[5] Grupo de Investigación en Tecnología para la Manufactura, Universidad Autónoma de Occidente, Santiago de Cali 760035, Colombia
[6] AIMPLAS, Gustave Eiffel 4 (València Parc Tecnològic), Paterna 46980, España
* Correspondence: edgar.mosquera@correounivalle.edu.co (E.M.-V.); jesus.diosa@correounivalle.edu.co (J.E.D.)

The figures S1-S4 show the Nyquist and Bode plots obtained from the impedance spectroscopy experiments (|Z| vs. frequency and ϕ vs. frequency, where |Z| and ϕ represent the impedance magnitude and phase, respectively). Experimental data are plotted with black dots, red lines represent the fitting curves obtained by modeling the impedance behavior to the equivalent electrical circuit proposed in the manuscript.

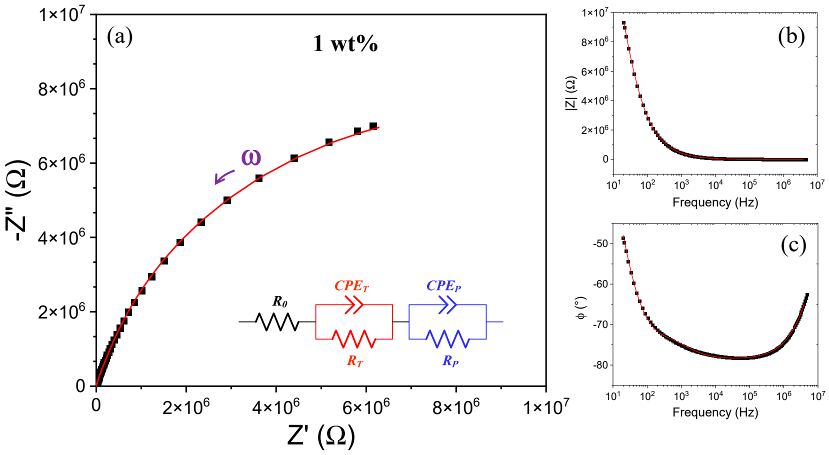

Figure S1. (**a**) Nyquist and (**b**,**c**) Bode plots for the TPU plate with 1 wt% NWCNT loading.

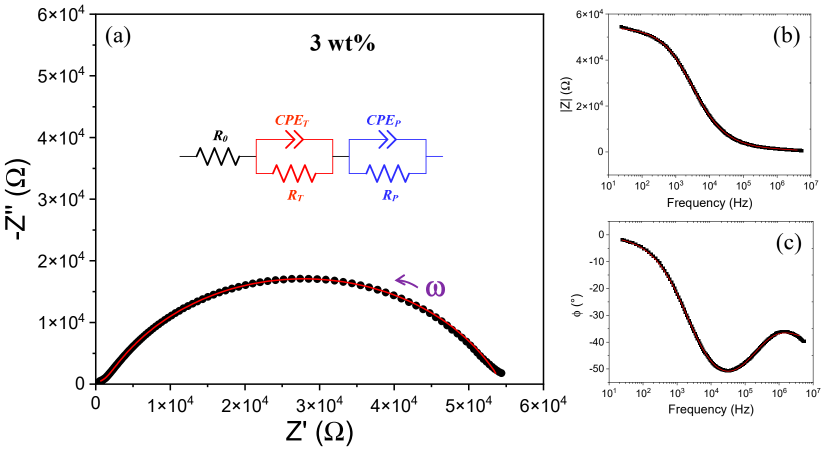

Figure S2. (**a**) Nyquist and (**b**,**c**) Bode plots for the TPU plate with 3 wt% NWCNT loading.

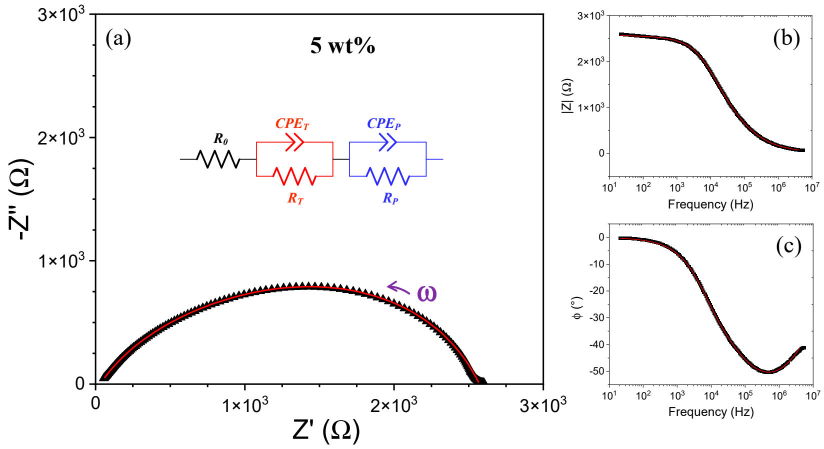

Figure S3. (a) Nyquist and (b, c) Bode plots for the TPU plate with 5 wt% NWCNT loading.

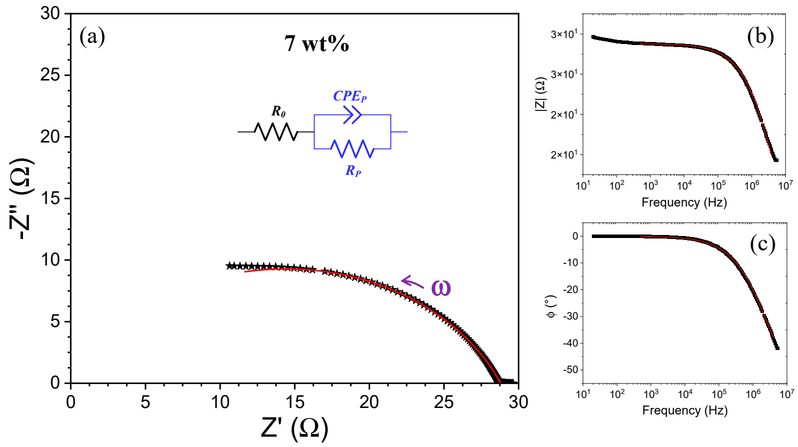

Figure S4. (**a**) Nyquist and (**b**,**c**) Bode plots for the TPU plate with 7 wt% NWCNT loading.

The fitting parameters obtained for each equivalent circuit are listed in Table S1. R_x and CPE_x (x = 0, T [tunneling] and P [percolation]) represent the resistor and constant phase elements, respectively. In the case of the CPE, it is described by the following equation:

$$CPE = R^{\frac{1-n}{n}} Q^{\frac{1}{n}}$$

where n is an empirical constant (n = CPE_x-P), Q corresponds to a pseudocapacitance (Q = CPE_x-T) and R is the resistor element which is in parallel to the CPE. In addition, Table S1 also contains the calculated values of conductivity for each resistor element.

Table S1. List of the electrical parameters obtained by an IS analysis on the TPU/MWCNT plates under study.

	1 wt%			3 wt%	
	Value	Error		Value	Error
R_0 (Ω)	85.4	0.8	R_0 (Ω)	80	20
CPE_T-T (F)	8.4×10^{-9}	0.8×10^{-9}	CPE_T-T (F)	4.8×10^{-8}	0.8×10^{-8}
CPE_T-P	0.87	0.02	CPE_T-P	0.59	0.01
R_T (Ω)	8×10^4	2×10^4	R_T (Ω)	1250	30
CPE_P-T (F)	1.19×10^{-9}	0.02×10^{-9}	CPE_P-T (F)	1.86×10^{-8}	0.01×10^{-8}
CPE_P-P	0.876	0.003	CPE_P-P	0.7268	0.0008
R_P (Ω)	1.80×10^7	0.02×10^7	R_P (Ω)	5.316×10^4	0.006×10^4
L (cm)	0.1606	0.0001	L (cm)	0.1772	0.0001
L/A (cm^{-1})	0.269	0.001	L/A (cm^{-1})	0.294	0.001
σ_0 ($S \cdot cm^{-1}$)	3.16×10^{-3}	0.04×10^{-3}	σ_0 ($S \cdot cm^{-1}$)	3.5×10^{-3}	0.6×10^{-3}
σ_T ($S \cdot cm^{-1}$)	3.2×10^{-6}	0.7×10^{-6}	σ_T ($S \cdot cm^{-1}$)	2.34×10^{-4}	0.07×10^{-6}
σ_P ($S \cdot cm^{-1}$)	1.50×10^{-8}	0.02×10^{-8}	σ_P ($S \cdot cm^{-1}$)	5.53×10^{-6}	0.03×10^{-6}

	5 wt%			7 wt%	
	Value	Error		Value	Error
R_0 (Ω)	29.9	0.2	R_0 (Ω)	--	0.01
CPE_T-T (F)	7.6×10^{-8}	0.3×10^{-8}	CPE_P-T (F)	1.85×10^{-7}	0.04×10^{-7}
CPE_T-P	0.87	0.01	CPE_P-P	0.729	0.001
R_T (Ω)	1040	70	R_P (Ω)	28.80	0.02
CPE_P-T (F)	2.03×10^{-7}	0.05×10^{-7}			
CPE_P-P	0.665	0.008			
R_P (Ω)	1510	70			
L (cm)	0.1808	0.0001	L (cm)	0.1850	0.0001
L/A (cm^{-1})	0.301	0.001	L/A (cm^{-1})	0.308	0.001
σ_0 ($S \cdot cm^{-1}$)	1.01×10^{-2}	0.01×10^{-2}	σ_0 ($S \cdot cm^{-1}$)	--	--
σ_T ($S \cdot cm^{-1}$)	2.9×10^{-4}	0.2×10^{-4}	σ_T ($S \cdot cm^{-1}$)	--	--
σ_P ($S \cdot cm^{-1}$)	2.0×10^{-4}	0.1×10^{-8}	σ_P ($S \cdot cm^{-1}$)	1.072×10^{-2}	0.005×10^{-2}

Figure S5. illustrates how we estimate the DC conductivity for each TPU plates by applying the Jonscher's power law at low frequency:

$$\sigma_{AC} = \sigma_{DC} + A\omega^s$$

where A is a pre-exponential factor, s is an exponent that helps to fit the frequency dependence of the AC conductivity (σ_{AC}), and σ_{DC} is the DC conductivity of the system.

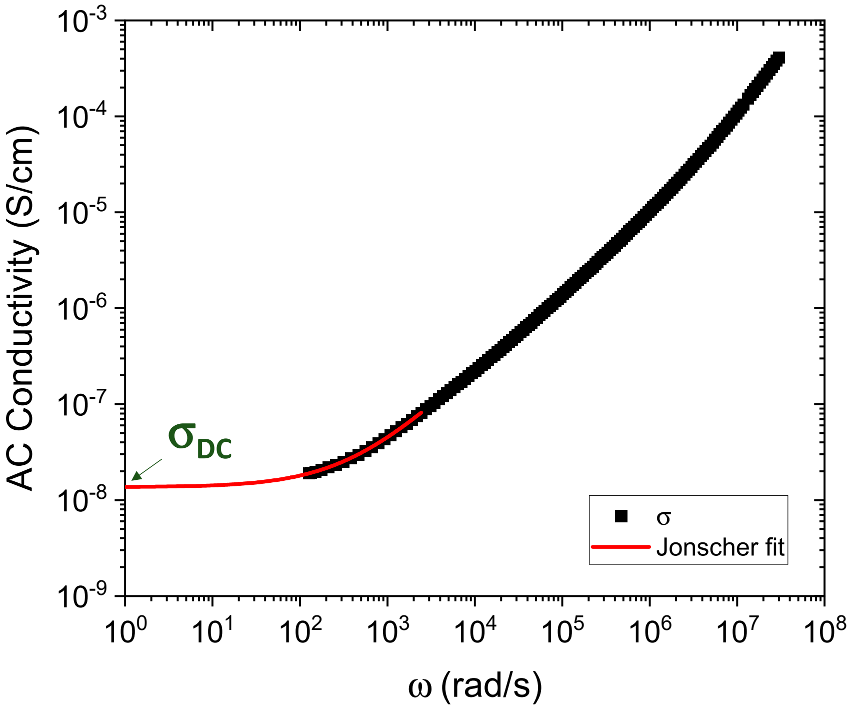

Figure S5. Plot of AC conductivity as a function of the angular frequency for the TPU/MWCNT plates with 1 wt% .
